# Statement of Retraction: Protein disulfide-isomerase A3 knockdown attenuates oxidized low-density lipoprotein-induced oxidative stress, inflammation and endothelial dysfunction in human umbilical vein endothelial cells by downregulating activating transcription factor 2

**DOI:** 10.1080/21655979.2025.2491958

**Published:** 2025-04-23

We, the Editors and Publisher of the journal *Bioengineered*, have retracted the following article:

Jia, J., Wang, Y., Huang, R., Du, F., Shen, X., Yang, Q., & Li, J. (2022). Protein disulfide-isomerase A3 knockdown attenuates oxidized low-density lipoprotein-induced oxidative stress, inflammation and endothelial dysfunction in human umbilical vein endothelial cells by downregulating activating transcription factor 2. *Bioengineered, 13*(1), 1436–1446. https://doi.org/10.1080/21655979.2021.2018980

Since publication, significant concerns have been raised about the integrity of the data and reported results in the article. Upon reviewing the original data and supporting documentation from the authors, significant concerns about the integrity of the data and results remain. As verifying the validity of published work is core to the integrity of the scholarly record, we are therefore retracting the article. The corresponding author listed in this publication has been informed.

We have been informed in our decision-making by our editorial policies and the COPE guidelines.

The retracted article will remain online to maintain the scholarly record, but it will be digitally watermarked on each page as ‘Retracted’.

